# Air-stable ambipolar field-effect transistor based on a solution-processed octanaphthoxy-substituted tris(phthalocyaninato) europium semiconductor with high and balanced carrier mobilities†
†Electronic supplementary information (ESI) available: Details of the syntheses, film-preparation procedure, structural characterization data and additional physical characterization data. See DOI: 10.1039/c4sc03492a



**DOI:** 10.1039/c4sc03492a

**Published:** 2014-12-10

**Authors:** Xia Kong, Xia Zhang, Dameng Gao, Dongdong Qi, Yanli Chen, Jianzhuang Jiang

**Affiliations:** a Shandong Provincial Key Laboratory of Fluorine Chemistry and Chemical Materials , School of Chemistry and Chemical Engineering , University of Jinan , Jinan 250022 , China . Email: chm_chenyl@ujn.edu.cn ; Fax: +86 0531 8973 6150; b Beijing Key Laboratory for Science and Application of Functional Molecular and Crystalline Materials , Department of Chemistry , University of Science and Technology Beijing , Beijing 100083 , China . Email: jianzhuang@ustb.edu.cn ; Fax: +86 0010 6233 2462

## Abstract

Simple solvent vapor annealing over QLS film-based OFET devices fabricated from (Pc)Eu[Pc(ONh)_8_]Eu[Pc(ONh)_8_] led to a high and balanced ambipolar performance that has never been observed for small molecule single-component-based solution processed devices, with mobilities of 1.71 and 1.25 cm^2^ V^–1^ s^–1^ for holes and electrons, respectively, under ambient conditions.

## Abstract 2

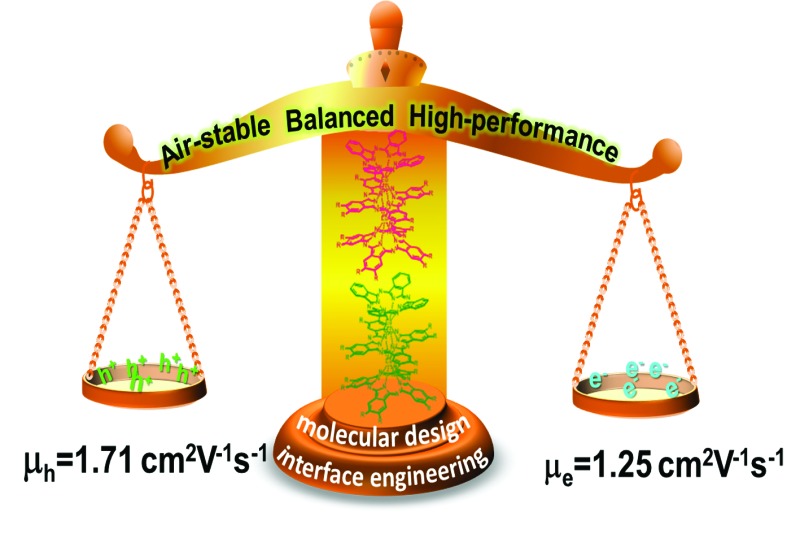
Simple solvent vapor annealing over QLS film-based OFET devices fabricated from (Pc)Eu[Pc(ONh)_8_]Eu[Pc(ONh)_8_] led to a high and balanced ambipolar performance.

## 

Since the first demonstration of ambipolar charge transport in a bilayer organic thin-film transistor of an organic semiconductor,^1^ significant progress has been made towards their practical application in ultra-low-cost, large-area complementary integrated circuits without necessarily requiring micropatterning of the individual p- and n-channel semiconductors.^2^ In particular, for the purpose of understanding the structure–functionality relationship, small molecule-based ambipolar organic semiconductors have attracted a wide range of research interest. In 1990, organic field-effect transistors (OFETs) fabricated from bis(phthalocyaninato) lanthanide compounds (Pc)M(Pc) (M = Tm, Lu) with a narrow band gap were found to show ambipolar characteristics with a hole mobility of 10^–3^ to 10^–4^ cm^2^ V^–1^ s^–1^ and an electron mobility of 10^–4^ to 10^–5^ cm^2^ V^–1^ s^–1^ in vacuum, revealing the ambipolar nature of a single-organic component for the first time.^3^ As anticipated, this study was followed by extensive investigations with the disclosing of the ambipolar nature for polymers,^4^ oligomers,^5^ and in particular a series of small molecules including acene derivatives with a wide band gap, *E*_gap_ > 1.8 eV.^6^ Among which, ultrapure rubrene single crystal-based OFET devices appear to present the best result with a hole mobility of 43 cm^2^ V^–1^ s^–1^ and an electron mobility of 0.81 cm^2^ V^–1^ s^–1^ in vacuum, despite the non-balanced mobilities between the two carriers mainly due to the mismatch between the standard electrodes (Au, Ag, or Al) and the LUMO level of rubrene.^7^ Modification of the acene backbone *via* silylethynylation seems to improve the balance, resulting in a hole mobility of 0.22 cm^2^ V^–1^ s^–1^ and an electron mobility of 1.1 cm^2^ V^–1^ s^–1^ also in vacuum, for the chemical vapor deposition (CVD) film-based OFET devices fabricated from silylethynylated *N*-heteropentacene.^8^ However, when tested in ambient air, the electron mobility significantly decreased to the range of 10^–3^ cm^2^ V^–1^ s^–1^ due to electron trapping by oxygen or water. To the best of our knowledge, solution processed small molecule-based ambipolar OFETs, in particular those with an air-stable nature, still remain rare, with the best result achieved by a (*p*-fluoro)phenoxy-substituted tris(phthalocyaninato) europium semiconductor with a hole mobility of 0.24 cm^2^ V^–1^ s^–1^ and an electron mobility of 0.042 cm^2^ V^–1^ s^–1^.^9^ For the purpose of further improving the device performance, in particular the balance between two carrier mobilities, both phthalocyanine and porphyrin ligands were simultaneously incorporated into a tris(tetrapyrrole) metal skeleton.^10^ This, however, induced a significant decrease in the charge mobilities despite the effective improvement in the balance between the two carrier mobilities.

On the other hand, the solvent vapor annealing (SVA) method using high boiling-point marginal solvent (instead of good and poor solvent) is well known to be able to effectively increase molecular ordering and orientation, and in turn, the crystallinity of a preformed thin film.^11^ As a consequence, in recent years this technique has been employed to improve the OFET performance of solution processed organic semiconductors of single-polarity. In 2006, by utilizing the SVA technique, the hole mobility of a triethylsilylethynyl anthradithiophene p-type semiconductor was effectively increased from 0.002 cm^2^ V^–1^ s^–1^ for the solution-processed film to 0.2 cm^2^ V^–1^ s^–1^ for the SVA film.^12^ Nevertheless, the mobility of the electrons in 1,2-dichloroethane (DCE)-vapor-annealed devices fabricated from n-type dialkyl-substituted-dicyanoperylene tetracarboxylic diimide derivatives was also effectively improved to a large degree from 0.02 to 0.5 cm^2^ V^–1^ s^–1^.^11^ However, to the best of our knowledge, this SVA method has not yet been employed to small molecule-based ambipolar organic semiconductor-based OFET devices.

In the present paper, we describe the design and preparation of a novel heteroleptic tris(phthalocyaninato) europium complex with an unsymmetrical triple-decker molecular structure, (Pc)Eu[Pc(ONh)_8_]Eu[Pc(ONh)_8_] (**1**) [Pc = unsubstituted phthalocyanine; Pc(ONh)_8_ = 2,3,9,10,16,17,23,24-octanaphthoxy phthalocyanine], Scheme 1. The electrochemical study reveals its potential air stability and balanced ambipolar organic semiconductor nature,^2*c*^ which is indeed verified by the performance, despite not being high, of the solution processed quasi-Langmuir–Shäfer (QLS) film-based OFET devices fabricated from this compound. Nevertheless, simple solvent annealing over the QLS films using *o*-dichlorobenzene (DCB) induces a significant improvement in the device performance with a good on/off ratio of 10^6^ and in particular, high and balanced carrier mobilities of 1.71 cm^2^ V^–1^ s^–1^ for holes and 1.25 cm^2^ V^–1^ s^–1^ for electrons that have never been reported for small molecule single-component-based OFET devices.

**Scheme 1:**
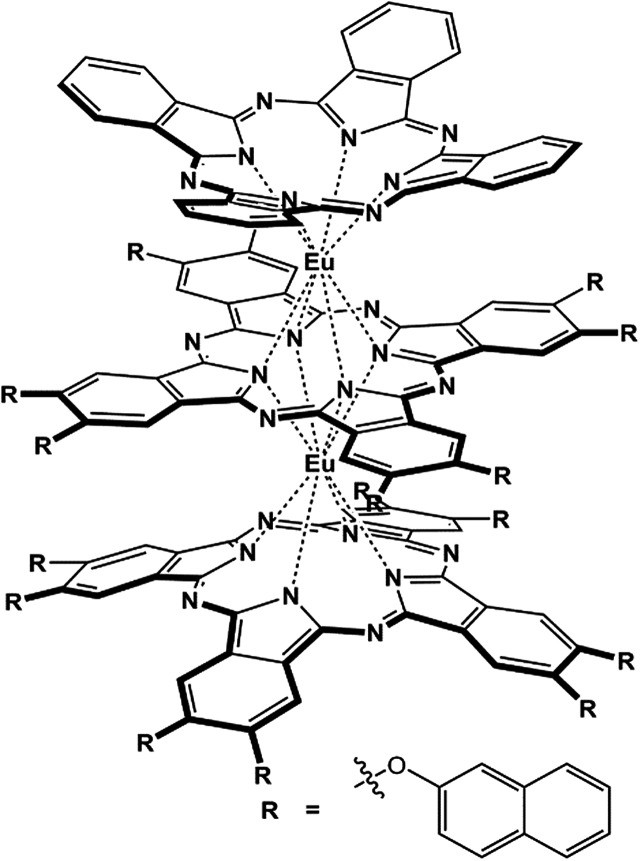
Schematic molecular structure of the tris(phthalocyaninato) europium triple-decker complex (Pc)Eu[Pc(ONh)_8_]Eu[Pc(ONh)_8_] (**1**).

The heteroleptic tris(phthalocyaninato) europium triple-decker complex (Pc)Eu[Pc(ONh)_8_]Eu[Pc(ONh)_8_] (**1**) was prepared following published procedures^9,13^ and characterized using MALDI-TOF mass spectrometry and a range of spectroscopic methods including ^1^H NMR, Fig. S1 and S2.† On the basis of previous results,^9,10,13^ in the present case eight naphthoxy substituents with a slight electron-withdrawing nature were introduced onto the periphery of the bottom and middle phthalocyanine ligands in the triple-decker molecule to tune the HOMO and in particular the LUMO energy level, towards enhancing the electron transport as well as the air-stability of the corresponding organic semiconductor.^14^ Nevertheless, the unsymmetrical structure of the target heteroleptic triple-decker compound isolated, in combination with the effect of the bulky naphthoxy substituents, also ensures its good solubility in common organic solvents and in turn its good solution processability.^13^ Most importantly, the design and preparation of such a heteroleptic tris(phthalocyaninato) europium compound involving different tetrapyrrole ligands is actually expected to afford an ambipolar organic semiconductor with balanced carrier mobility between electrons and holes as mentioned above.^10^

Differential pulse voltammetry (DPV) measurement of (Pc)Eu[Pc(ONh)_8_]Eu[Pc(ONh)_8_] (**1**) in CH_2_Cl_2_ reveals a couple of one-electron redox couples with the first oxidation and first reduction potential at +0.63 and –0.44 V (*vs.* SCE), Fig. S3 and Table S1.† Both the HOMO and LUMO energies at –5.07 and –4.00 eV thus derived for this triple-decker compound match well with the work function of the gold electrode at –5.1 eV and are located in the energy range required for good p- and in particular air-stable n-type organic semiconductors, respectively, ensuring the simultaneous facilitation of both the hole and electron injections from the Au electrodes. This in turn suggests the potential of this triple-decker compound for use in air-stable ambipolar OFET devices with good performance.

Ordered multilayers of (Pc)Eu[Pc(ONh)_8_]Eu[Pc(ONh)_8_] (**1**) were prepared using a solution-based QLS method.^15^ The morphology of the triple-decker QLS film was characterized by atomic force microscopy (AFM) and scanning electron microscopy (SEM), Fig. 1A and B. The images show small domains of approximately 40–60 nm in size, with some gaps and cracks between aggregate domains. The out-of-plane (OOP) X-ray diffraction pattern (XRD) of the QLS film in the low-angle range exhibits one sharp diffraction peak at 2.19 nm (2*θ* = 4.05°), Fig. 2A, which corresponds to the thickness of one layer of QLS film, suggesting a layered structure for this QLS film.^16^ Polarized UV-vis spectroscopy was employed to detect the orientation (dihedral angle between the Pc rings and the surface of the substrate) of the Pc rings in the film,^17^ revealing an “edge-on” conformation of the triple-decker molecules in the film on the substrate with a dihedral angle of 52.6° and the formation of J-aggregates of the triple-decker molecules in the film, Fig. S4A and Table S2† and inset of Fig. 2A. This is in line with the calculated result based on the simulated triple-decker molecular dimension (2.86 nm, Fig. S5†)^13,18^ and the above-mentioned OOP XRD result (2.19 nm), with the orientation angle of 50.0°. Furthermore, the triple-decker compound (Pc)Eu[Pc(ONh)_8_]Eu[Pc(ONh)_8_] (**1**) displays a Q band at 655 nm in CHCl_3_ solution, Fig. S6,† which red-shifts to 662 nm in the QLS film, confirming the formation of J-aggregates and indicating the strong interaction between neighboring molecules.^19^

**Fig. 1:**
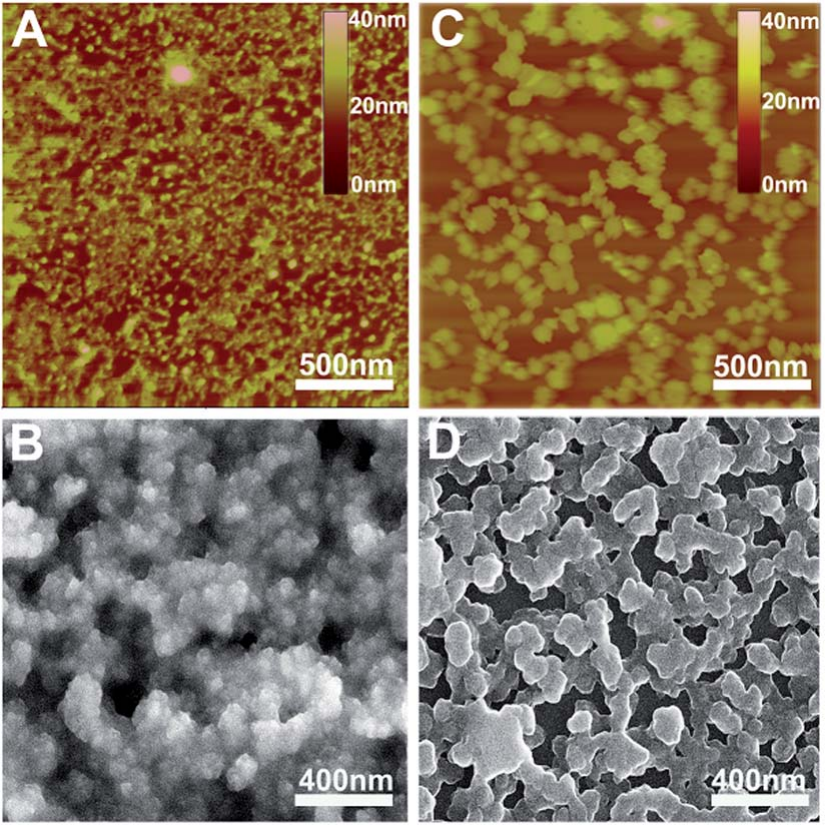
AFM and SEM images of the pristine QLS film (A and B) and the SVA film (C and D) of **1**.

**Fig. 2:**
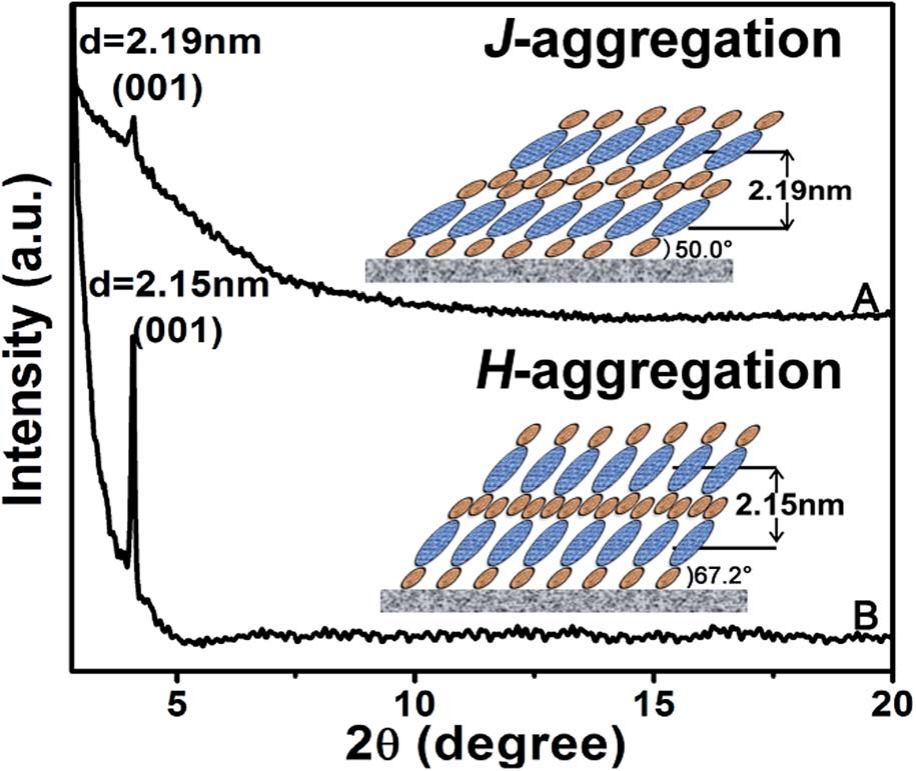
XRD patterns of the pristine QLS film (A) and the SVA film (B) of **1**. The insets are schematic packing modes of the triple-decker compound **1** in the QLS and the SVA film, respectively.

As exemplified in Fig. S7,† the OFET device fabricated from **1** on a hexamethyldisilazane (HMDS)-treated SiO_2_/Si substrate using the QLS technique with a bottom-gate top-contact configuration showed typical ambipolar (both p-channel and n-channel) characteristics in air. The carrier mobility (*μ*) was calculated using the saturation region transistor equation, *I*_ds_ = (*W*/2*L*)*μC*_0_(*V*_G_ – *V*_T_),^2^ where *I*_ds_ is the source–drain current, *V*_G_ the gate voltage, *C*_0_ the capacitance per unit area of the dielectric layer, and *V*_T_ the threshold voltage.^20^ In air, the device presents a carrier mobility for holes of 2.16 × 10^–6^ cm^2^ V^–1^ s^–1^ (*V*_ds_, –100 V), Fig. S7A.† Nevertheless, under ambient conditions this device simultaneously displays a carrier mobility for electrons of 3.15 × 10^–6^ cm^2^ V^–1^ s^–1^ (*V*_ds_, 100 V), Fig. S7B.† These results clearly reveal the air-stable ambipolar nature of the OFET device fabricated from the heteroleptic tris(phthalocyaninato) europium compound with balanced carrier mobilities between holes and electrons despite their relatively low values.^9,10,13^ In line with this result, the current on/off ratios, located in the range of *ca.* 10^2^ for both the p-and n-channel transports in the ambipolar OFET device, are also not good for the QLS film-based devices of **1**, Table S3.† This, however, seems untrue for the threshold voltages (*V*_T_) with values of –16 and +2 V for holes and electrons, respectively, obtained for the QLS film-based OFET device. In comparison with the ambipolar performance of the (Pc)Eu[Pc(OPh)_8_]Eu[Pc(OPh)_8_] QLS film-based devices reported previously by this group with mobilities of 0.68 cm^2^ V^–1^ s^–1^ for electrons and 0.014 cm^2^ V^–1^ s^–1^ for holes,^13^ indeed lower carrier mobilities for both holes and electrons were obtained for the (Pc)Eu[Pc(ONh)_8_]Eu[Pc(ONh)_8_] QLS film-based OFETs in the present case. This seems strange at first glance, but could be easily rationalized on the basis of the formation of different aggregates in QLS films with a H-aggregation mode revealed for (Pc)Eu[Pc(OPh)_8_]Eu[Pc(OPh)_8_]^13^ and a J-aggregation mode for (Pc)Eu[Pc(ONh)_8_]Eu[Pc(ONh)_8_] found in the present work on the basis of a series of spectroscopic, and in particular, XRD analysis results.

To enhance the performance of the device fabricated from this triple-decker compound, the pristine QLS films of **1** were simply treated by solvent vapor annealing under an atmosphere saturated with the marginal DCB vapor at 100 °C for 30 min by releasing 20 μL of solvent into the Petri dish (diameter: 9.5 cm, height: 1.5 cm, volume: 106.3 cm^3^), Fig. S8,† affording solvent vapor annealed (SVA) films. The morphology of the triple-decker SVA film was observed using AFM and SEM to show a typical two-dimensional wafer-like structure with more uniform grain crystallites, approximately 100–150 nm in diameter, and a decreased grain boundary compared to those of the pristine QLS film, Fig. 1C and D, revealing a highly improved film structure and morphology after the annealing process using DCB solvent likely due to the significant lowering of the activation energy for the rearrangement of soluble π-conjugated molecules.^11,12^ The increase in the grain size and the decrease in the grain boundary would be beneficial to the charge transport in such a SVA film relative to its pristine QLS film. The out-of-plane (OOP) XRD pattern in the low-angle range of the SVA film exhibits one strong and sharp diffraction peak at 2.15 nm (2*θ* = 4.10°), Fig. 2B, which corresponds to the thickness of one layer of SVA film, suggesting a regular layered structure for this film.^16^ Polarized UV-vis spectroscopic measurements reveal a slipped co-facial H-type stacking mode of the triple-decker molecules in the film on the substrate due to a dihedral angle of 67.2°, Fig. S4B and Table S2† and inset of Fig. 2B. As a consequence, the *d*-spacing of 2.15 nm in the SVA film revealed from OOP XRD measurements does not correspond with that calculated according to the simulated triple-decker molecular dimension (2.86 nm)^13,18^ and the polarized UV-vis result (67.2°), 2.64 nm. Such an obvious decrease (*ca.* 18%) of *d*-spacing in the SVA film indeed seems strange at first glance, however it could be rationalized on the basis of the increased interlayer interaction through effective side naphthoxy-moiety interdigitation between the neighbouring triple-decker molecules in the out-of-plane direction upon DCB vapor annealing.^21^ It is noteworthy that the (001) diffraction peak with increased intensity and sharpening in the low-angle region for the SVA film relative to that for the pristine QLS film of **1** clearly indicates the obviously improved molecular ordering and enhanced crystallinity in the SVA film over that in the QLS film, Fig. 2.^11,22^ In addition, in contrast to the QLS film of **1**, solvent annealing using DCB induces a dramatic blue-shift of the Q band from 655 nm for **1** in CHCl_3_ solution to 642 nm for the SVA film, Fig. S6,† indicates the presence of a strong face-to-face intermolecular stacking interaction and formation of H-aggregates between the π-conjugated triple-decker molecules.^19,23^ As a consequence, simple solvent vapor annealing of the (Pc)Eu[Pc(ONh)_8_]Eu[Pc(ONh)_8_] QLS films not only induces significant improvement over the molecular packing and ordering as well as the film-crystallinity, but also achieves successful fine control of the aggregation mode from J-type in the QLS films to H-type in the SVA films. The strong intramolecular-stacking in the triple-decker molecule together with intense intermolecular face-to-face interaction in the H-aggregates of the SVA film is believed to provide the electrons (or holes) with an extensive area for delocalization.^24^

As exemplified in Fig. 3 and Table S3,† the OFET devices fabricated from the SVA films of **1** on the HMDS-treated SiO_2_/Si substrates with a bottom-gate top-contact configuration also showed typically ambipolar (both p- and n-channel) but enhanced performance in comparison with the QLS film counterparts when measured in air, as exemplified by good current on/off ratio as high as 10^6^. In particular, these devices present high and balanced carrier mobilities of 1.71 cm^2^ V^–1^ s^–1^ for holes (at *V*_ds_ = –20 V) and 1.25 cm^2^ V^–1^ s^–1^ for electrons (at *V*_ds_ = 20 V), Fig. 3 and Table S3.† It should also be pointed out that among the 32 totally examined OFETs, over 70% of the devices exhibit carrier mobilities of over 1 cm^2^ V^–1^ s^–1^ with an average value for electrons of 1.25 ± 0.1 cm^2^ V^–1^ s^–1^ and for holes of 1.75 ± 0.25 cm^2^ V^–1^ s^–1^, respectively, Fig. S9.† To the best of our knowledge, this result represents the ambipolar performance in terms of the highest carrier mobility values for holes and electrons measured simultaneously, and in particular, the balance between them, among solution-processed small-molecule single-component-based OFET devices under ambient conditions.^5,23^ Nevertheless, even after 2 months stored under ambient conditions, the devices fabricated from the SVA films of this triple-decker compound still exhibit high and balanced electron and hole mobilities in the order of 1.0 cm^2^ V^–1^ s^–1^ (*μ*_h_ = 1.11 cm^2^ V^–1^ s^–1^ and *μ*_e_ = 1.04 cm^2^ V^–1^ s^–1^) with the current on/off ratio remaining unchanged for both n- and p-channel operations, Fig. S10 and Table S3,† indicating good device stability in air. It is worth noting that in the low source–drain biased voltage, the contact effect was observed for not only the electron but also for the hole transport in the present work, although almost no Mott–Schottky barrier exists for the hole injection due to good matching between the HOMO (–5.07 eV) of (Pc)Eu[Pc(ONh)_8_]Eu[Pc(ONh)_8_] and the Fermi level of the Au electrode (–5.1 eV). As a result, the contact effect observed for both electron and hole transport in the low source–drain voltage range should be attributed mainly to the injection barriers from charge carrier trap states within the solution-based organic semiconductor film and at the organic semiconductor–metal interface.^25^ However, it should again be pointed out that despite the relatively large injection barriers, surprisingly efficient electron and hole injection from gold into the semiconductor layer still becomes possible along with the increase in the source–drain voltage, resulting in high carrier mobilities for both holes and electrons that can be extracted from the saturation regime. This result seems to indicate that the transfer and output characteristics for the ambipolar triple-decker organic semiconductor have not been affected by the contact resistance in the saturation regime, since the gate field applied induces additional charges to fill the deep trap states, Fig. 3, which in turn helps the hole and electron injection from the gold electrodes into the organic semiconducting layer.

**Fig. 3:**
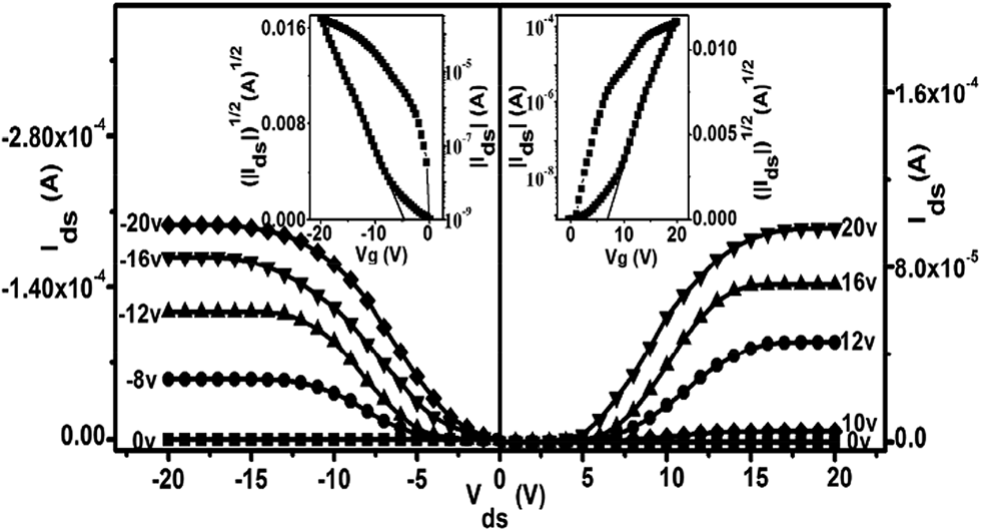
Output characteristics (*I*_ds_ *versus V*_ds_) and (insets) transfer characteristics (|*I*_ds_|^1/2^ *versus V*_G_) for the ambipolar OFET device based on the SVA film of **1** deposited on a HMDS-treated SiO_2_/Si (300 nm) substrate with Au top contacts measured in air.

As can be expected significant improvement, particularly in terms of the balance between the threshold voltages (*V*_T_) for holes and electrons, has also been achieved after solvent annealing of the QLS films of **1**. The fairly low and balanced threshold voltages for holes and electrons, –5 and +9 V, respectively, obtained for the SVA film-based OFET devices of **1**, in combination with high charge mobilities and a good on/off ratio, ensure the application potential of this air-stable ambipolar organic semiconductor in low-power nano-electronics.^26^ In addition, the operating voltage range applied decreases from 0–±80 V for QLS film-based OFETs to 0–±20 V for the SVA film-based devices, indicating the effect of the molecular alignment and ordering improvement by solvent vapor annealing on the device behavior. Nevertheless, in the present case the high and balanced carrier mobilities for holes and electrons have been recorded over both a source–drain voltage range of (*V*_ds_ = 0–±20 V) and a gate voltage range of (*V*_G_ = 0–±20 V), which are quite small voltage ranges when compared with the usual larger *V*_ds_ and *V*_G_ voltage ranges, higher than 0–±20 V, previously reported for organic thin film transistors.^27^ Furthermore, both the output and transfer curves for the (Pc)Eu[Pc(ONh)_8_]Eu[Pc(ONh)_8_] ambipolar OFET devices at a low gate bias do not show a current increase in a superlinear manner at high drain bias, Fig. 3 and S7,† likely due to the simultaneous considerably lower injection barrier for holes and electrons,^28^ ensuring their future practical applications. Actually, careful inspection of the OFET devices reported thus far reveals that in comparison with single-polar OFET devices, there are many more examples of ambipolar OFETs with the superlinear feature observed in the output curves at a gate bias of 0 V,^29^ due to the large carrier barrier for either holes and/or electrons because of the lack of a simultaneous match of the work function of the metal electrodes with the HOMO and/or LUMO level of the ambipolar semiconductor.^25a,30^ As a result, for the purpose of effectively eradicating the superlinear property of the ambipolar OFET devices, various methods have been developed to simultaneously lower the injection barrier for holes and electrons, including varying the work function of the electrodes,^31^ employing a two-component organic heterostructure,^1,32^ or utilizing a low bandgap organic semiconductor.^5a,33^ In the present case, the HOMO level of (Pc)Eu[Pc(ONh)_8_]Eu[Pc(ONh)_8_] aligns well with the work function of the gold electrodes, ensuring an ohmic contact for hole injection from gold into the semiconductor layer. More interestingly, despite the mismatch between the work function of the Au electrode and the LUMO energy level of (Pc)Eu[Pc(ONh)_8_]Eu[Pc(ONh)_8_] at –4.00 eV (if this was the sole factor, an electron injection barrier as large as 1.1 eV in a zero-order vacuum alignment would be anticipated), an ohmic-like contact for electron injection still becomes possible due to the formation of interface dipoles^34^ at the Au/(Pc)Eu[Pc(ONh)_8_]Eu[Pc(ONh)_8_] interface, which also considerably lower the electron injection barrier, ensuring and also rationalizing the observation of non-superlinear transport for electrons. This is in line with those observed for good ambipolar OFET devices reported previously.^31–34^

## Conclusions

Briefly summarizing the above, a simple solvent vapor annealing approach using the high boiling-point marginal solvent DCB over QLS films fabricated from heteroleptic (Pc)Eu[Pc(ONh)_8_]Eu[Pc(ONh)_8_] with an unsymmetrical triple-decker molecular structure and bringing peripheral slightly electron-withdrawing naphthoxy substituents led to a more ordered molecular packing in the film, enabling an excellent ambipolar OFET device performance that has never been measured for small molecule single-component-based solution processed devices, with high and balanced mobilities of 1.71 and 1.25 cm^2^ V^–1^ s^–1^, low threshold voltages of –5 and +9 V for holes and electrons, respectively, and high on/off ratios of 10^6^. The present result will be helpful for the design and preparation of air-stable, high performance ambipolar OFET devices with potential application in ultra-low-cost, large-area complementary integrated circuits through the combination of molecular design and interface engineering.
