# Supplementary material for: Air-stable ambipolar field-effect transistor based on a solution-processed octanaphthoxy-substituted tris(phthalocyaninato) europium semiconductor with high and balanced carrier mobilities
Source: Chem Sci. 2014 Dec 10;6(3):1967–72. doi: 10.1039/c4sc03492a (PMC5495994; doi:10.1039/c4sc03492a)
Supplement: Supplementary file 1 [file SC-006-C4SC03492A-s001.pdf]

Electronic Supplementary Material (ESI) for Chemical Science.
This journal is © The Royal Society of Chemistry 2014

Electronic Supplementary Information (ESI) for

Air-stable ambipolar field-effect transistor based on a solution–processed octanaphthoxy-substituted tris(phthalocyaninato) europium semiconductor with high and balanced carrier mobilities

Xia Kong, [a] *Xia Zhang,* [a] *Dameng Gao,* [a] *Dongdong Qi,*[b] *Yanli Chen*[*a] *and Jianzhuang Jiang*[*b]

List of Contents

1. **Experimental Section**
2. **MALDI–TOF mass spectrum and ^{1}H NMR spectrum**
3. **DPV of (Pc)Eu[Pc(ONh)$_8$]Eu[Pc(ONh)$_8$]**
4. **PUV and UV spectrum for QLS film and SVA film**
5. **The schematic of molecular dimension**
6. **The preparation procedure of QLS and SVA film.**
7. **OFET for QLS film**
8. **The distribution diagram of devices mobilities**
9. **Stability Study**
10. **AFM images for the thickness of the pristine QLS film and SVA film**

Experimental Section

Measurements: ^{1}H NMR spectrum was recorded on a Bruker DPX 400 spectrometer in $CDCl_3$. Spectrum was referenced internally using the residual solvent resonance (δ = 7.26 for ^{1}H NMR) relative to $SiMe_4$. MALDI–TOF mass spectrum was taken on a Bruker BIFLEX III ultra-high-resolution Fourier transform ion cyclotron resonance (FT–ICR) mass spectrometer with alpha-cyano-4-hydroxycinnamic acid as matrix. Electrochemical measurement was carried out with a CHI760D voltammetric analyzer. The cell comprised inlets for a glassy carbon disk working electrode of 3.0 mm in diameter and a silver-wire counter electrode. The reference electrode was Ag/Ag^+ (0.01 mol dm^{-3}), which was connected to the solution by a Luggin capillary, whose tip was placed close to the working electrode. It was corrected for junction potentials by being referenced internally to the ferrocenium/ferrocene (Fc^+/Fc) couple [$E_{1/2}(Fc^+/Fc)$ = 0.50 V *vs.* SCE]. Typically, a 0.1 mol dm^{-3} solution of $[Bu_4N][ClO_4]$ in CH_2Cl_2 containing 0.5 mmol dm^{-3} of sample was purged with nitrogen for 5 min, then the voltammogram was recorded at ambient temperature. Electronic absorption spectra were recorded on a Hitachi U-4100 spectrophotometer. The X-ray diffraction experiment was carried out on a Bruker AXS D8 ADVANCE X-ray diffractometer. AFM image was collected under ambient conditions using the tapping mode with a NanoscopeIII/Bioscope scanning probe microscope from Digital instruments. Scanning electron microscopy (SEM) image was obtained using a JEOL JSM-6700F field-emission scanning electron microscopy. For SEM imaging, Au (1-2 nm) was sputtered onto the substrate to prevent charging effects and to improve the image clarity.

Chemicals: Anhydrous 1,2,4-trichlorobenzene(TCB), 1,8-diazabicyclo[5.4.0] undec-7-ene (DBU) were purchased from Aldrich. DMF was distilled from anhydrous $MgSO_4$ and *n*-pentanol was distilled from sodium. Dichloromethane for voltammetry study was freshly distilled from CaH_2 under an atmosphere of nitrogen. Column chromatography was carried out on silica gel (200–300 mesh) with the indicated eluents. All other reagents and solvents

were used as received. The compounds of $Eu(acac)_3 \cdot H_2O$ and 4,5-bis(naphthoxy)-1,2-dicyanobenzene, H_2Pc, $Eu[Pc(ONh)_8]_2$ were prepared according to the literature methods.[1-4]

Synthesis of (Pc)Eu[Pc(ONh)$_8$]Eu[Pc(ONh)$_8$]: A mixture of $Eu(acac)_3 \cdot H_2O$ (10 mg), H_2Pc (15 mg) and $Eu[Pc(ONh)_8]_2$ (50 mg) in TCB (3 mL) was refluxed for 5h under a slow stream of nitrogen. After being cooled to room temperature, 10 mL *n*-hexane was added to the mixture. The precipitate was filtered off, and then washed with *n*-hexane and methanol. The residue left was chromatographed on a silica gel column with $CHCl_3$ as eluent. Repeated chromatography followed by recrystallization from $CHCl_3$ and *n*-hexane gave pure compound as a blue powder.[5,6] Yield: 31 mg (53 %). ^{1}H NMR (400 MHz, $CDCl_3$, δ): 12.57 (s, 8 H, Pc-H_{α}), 10.00 (s, 8 H, Pc*-H_{α}), 9.55 (s, 8 H, Pc**-H_{α}), 9.48 (s, 8 H, Pc-$H_{\alpha2}$), 9.05-9.03(d, 8 H, Pc*-$H_{\beta1}$), 8.43-8.40(d, 8 H, Pc**-$H_{\beta1}$), 8.32-8.30(d, 8 H, Pc*-$H_{\beta2}$), 8.24-8.27(d, 8 H, Pc**-$H_{\beta2}$), 8.03-7.87 (m, 48 H, Pc*-Nh-$H_{\beta3}$, Pc**-Nh-$H_{\beta3}$, Pc*-Nh-$H_{\beta4}$, Pc**-Nh-$H_{\beta4'}$, Pc**-Nh-$H_{\beta5}$, Pc*-Nh-$H_{\beta5'}$), 7.61-7.56(m, 24 H, Pc*-Nh-$H_{\beta6}$, Pc**-Nh-$H_{\beta6'}$, Pc*-Nh-$H_{\beta7}$), 7.45-7.41(t, 8 H, Pc*-Nh-$H_{\beta7'}$); MALDI-TOF MS: an isotopic cluster peaking at m/z 4117, Calcd for $C_{256}H_{146}Eu_2N_{24}O_{16}$, $[M]^+$, 4118.

The molecule simulations: The molecule structure with all real frequencies are optimized at the level of B3LYP/6-311G(d)/SDD. All the calculations are carried out using Gaussian 09 D.01 (M. J. Frisch, Gaussian 09 (Version A.02), Gaussian, Inc.: Wallingford CT, 2009.), showing and measuring with PCMODEL (K. E. Gilbert, PCMODEL, "Serena Software BOX 3076", Bloomington, USA, 2004). The resulting molecular dimensions are shown in Fig. S5†.

The preparation of SVA film of the triple-decker: As shown in Fig. S8†, the preparation of the SVA film required a two-step procedure: (i) *The preparation of the pristine QLS film*: A chloroform solution (5.0 mL) of **1** ($\sim 1 \times 10^{-6}$ mol L^{-1}) was placed into a cylindrical glass container (diameter: 9.5 cm, height: 1.5 cm, volume: 106.3 cm^3), then water (65 mL) than the chloroform solution was slowly added onto the surface of the chloroform solution to keep the

path for evaporation of organic solvent. During solvent evaporation, triple-decker molecules gradually assembled to form some fine nanostructures at the $CHCl_3$/water interface and thus the densely packed film on the water surface until complete evaporation of $CHCl_3$. The film can be easily transferred from the water surface onto a glass or SiO_2/Si substrate by horizontal lifting, *i.e.* the slide was arranged horizontally and carefully lowered onto the film surface and then raised.[7] The process was repeated to obtain the required number of layers. Residual water on the substrates, between transfer steps and after the final transfer, was removed with a stream of N_2. (ii) *The preparation of the final SVA film*: In order to further improve film microstructure fabricated by QLS method, annealing of the QLS film was done at 100 °C under DCB vapor for 30 min by releasing 20μL of solvent in the Petri dish (diameter: 9.5 cm, height: 1.5 cm, volume: 106.3 cm^3), which resulted in a SVA film.[8] And the thickness of the films were obtained by measuring the depth of a scratch on the surface in the AFM as 120 nm for the QLS film and 106 nm for the SVA film, respectively, see Fig. S11†.

Fabrication and measurements of OFET device: OFET devices were fabricated on the HMDS-treated SiO_2/Si (300 nm thickness, capacitance C_0=10 nF/cm^2) substrates by evaporating gold electrodes onto both the SVA and the QLS films of **1** employing a shadow mask. These electrodes have a width (*W*) of 28.6 mm and a channel length (L) of 0.24 mm. The ratio of the width to length (*W/L*) of the channel was then 119. The drain-source current (I_{ds}) versus drain-source voltage (V_{ds}) characteristic was obtained with a Hewlett–Packard (HP) 4140B parameter analyzer at room temperature. Experimental data were analyzed using standard field-effect transistor equations: $I_{ds} = (W/2L)\mu C_0(V_G - V_T)^2$, where I_{ds} is the source-drain current, V_G is the gate voltage, C_0 is the capacitance per unit area of the dielectric layer, and V_T is the threshold voltage, and μ is the mobility in the saturation region.[9] A linear fit was applied in the saturation region of V_G *vs*. $(I_{ds})^{1/2}$ curve of each of the triple-deckers in order to calculate the mobility.

References

1. J. G. Stites, C. N. McCarty and L. L. Quill, *J. Am. Chem. Soc*., 1948, **70**, 3142.
2. M. J. Plater, A. Jeremiah and G. Bourhill, *J. Chem. Soc. Perkin Trans.,* 2002, **1**, 91.
3. S. Vajiravelu, L. Ramunas, G. J. Vidas, G. Valentas, J. Vygintas and S. Valiyaveettil, *J. Mater. Chem.,* 2009, **19**, 4268.
4. G. Lu, M. Bai, R. Li, X. Zhang, C. Ma, P. C. Lo, D. K. P. Ng and J. Jiang, *Eur. J. Inorg. Chem.,* 2006, **18**, 3703.
5. D. Li, H. Wang, J. Kan, W. Lu, Y. Chen and J. Jiang, *Org. Electron.,* 2013, **14**, 2582.
6. J. Kan, Y. Chen, D. Qi, Y. Liu and J. Jiang, *Adv. Mater.,* 2012, **24**, 1755.
7. Y. Chen, M. Bouvet, T. Sizun, Y. Gao, C. Plassard, E. Lesniewska and J. Jiang, *Phys. Chem. Chem. Phys.,* 2010, **12**, 12851.
8. D. Khim, K. Baeg, J. Kim, M. Kang, S. –H. Lee, Z. Chen, A. Facchetti, D.-Y. Kim and Y.-Y. Noh, *ACS Appl. Mater. Inter.,* 2013, **5**, 10745.
9. S. M. Sze, *Physics of Semiconductor Devices*, John Wiley &Sons, New York, 1981.

Figs. S1-S9 and Tables S1-S3

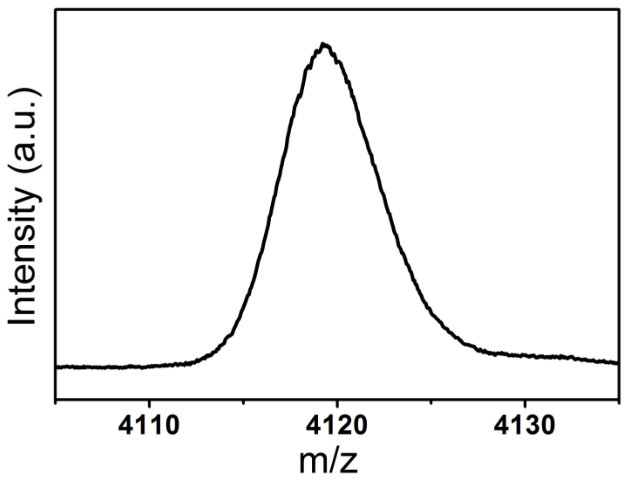

Fig. S1 Experimental isotopic pattern for the molecular ion of (Pc)Eu[Pc(ONh)$_8$]Eu[Pc(ONh)$_8$] (**1**) shown in the MALDI–TOF mass spectrum.

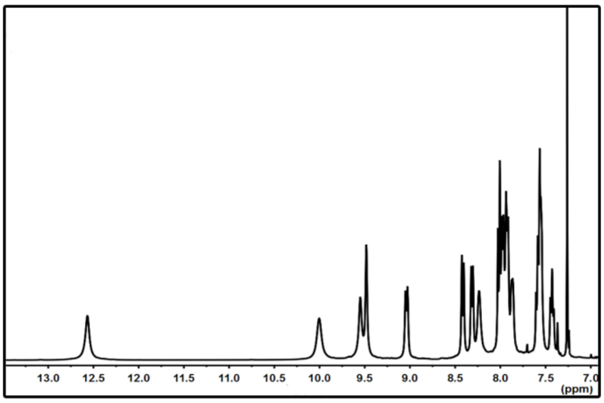

Fig. S2 ^{1}H NMR spectrum of (Pc)Eu[Pc(ONh)$_8$]Eu[Pc(ONh)$_8$] (**1**) in $CDCl_3$.

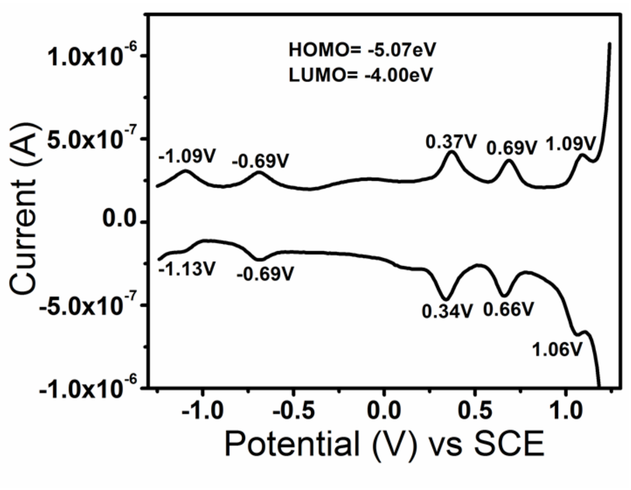

Fig. S3 Differential pulse voltammetry (DPV) of (Pc)Eu[Pc(ONh)$_8$]Eu[Pc(ONh)$_8$] (**1**) in CH_2Cl_2.

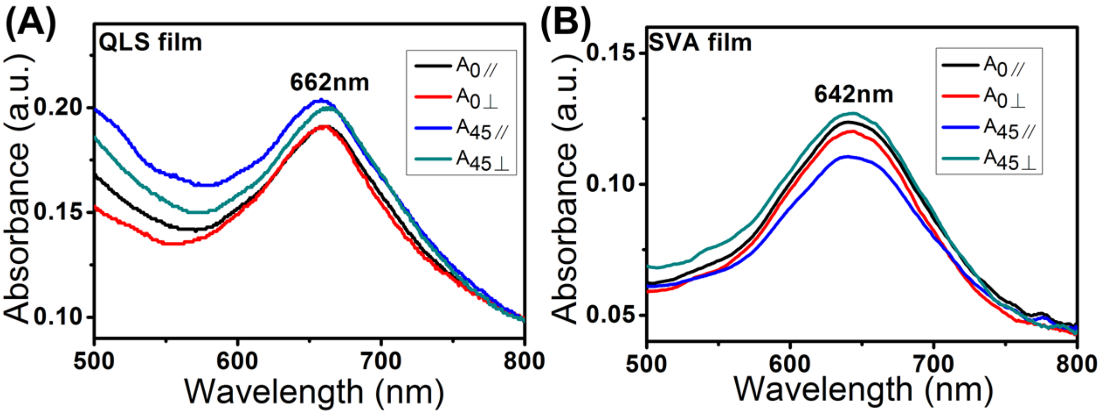

Fig. S4 Polarized UV–vis spectra for QLS film (A) and SVA film (B) of (Pc)Eu[Pc(ONh)$_8$]Eu[Pc(ONh)$_8$] (**1**). (0° and 45° represent the angle between the light and the normal of the substrate, respectively, while "A$_{//}$ and A$_{\perp}$" represent the absorbance for light polarized with the electric vector parallel and perpendicular to the dipping direction).

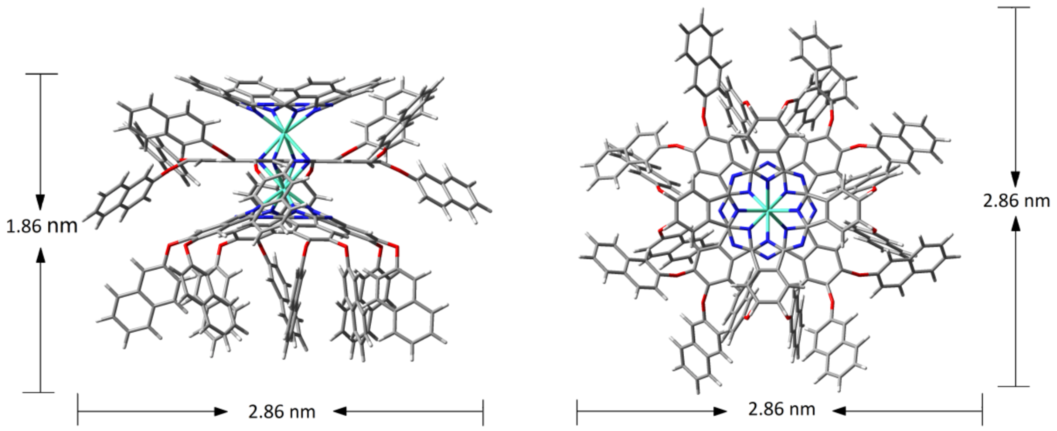

Fig. S5 The molecular dimension of (Pc)Eu[Pc(ONh)$_8$]Eu[Pc(ONh)$_8$] (**1**): 2.86 nm (width) × 1.86 nm (height) obtained using PCMODEL for windows Version 6.0, Serena Software.

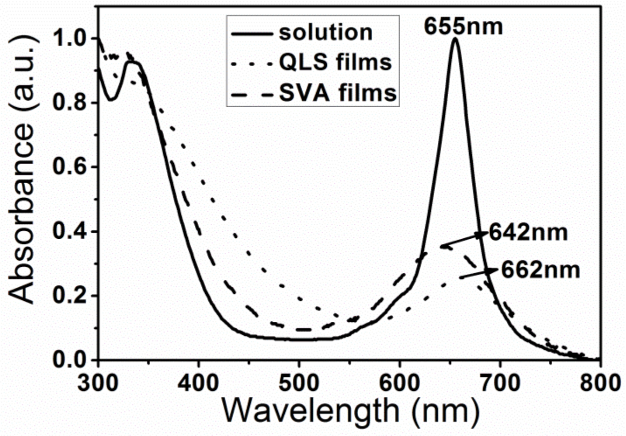

Fig. S6 Electronic absorption spectra of (Pc)Eu[Pc(ONh)$_8$]Eu[Pc(ONh)$_8$] (**1**) in dilute chloroform solution (black line), QLS film (dot line) and SVA film (dash line).

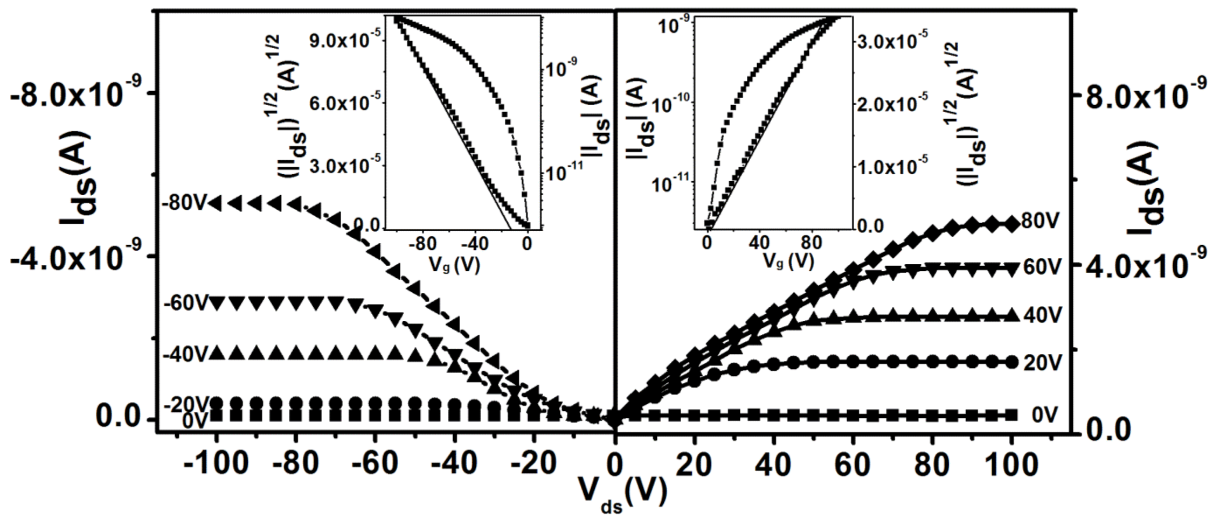

Fig. S7 Output characteristics (I_{ds} versus V_{ds}) and (insets) transfer characteristics ($|I_{ds}|^{1/2}$ versus V_G) of ambipolar OFET device based on the QLS film of (Pc)Eu[Pc(ONh)$_8$]Eu[Pc(ONh)$_8$] (**1**) deposited on HMDS-treated SiO_2/Si substrate with Au top contacts measured under ambient condition.

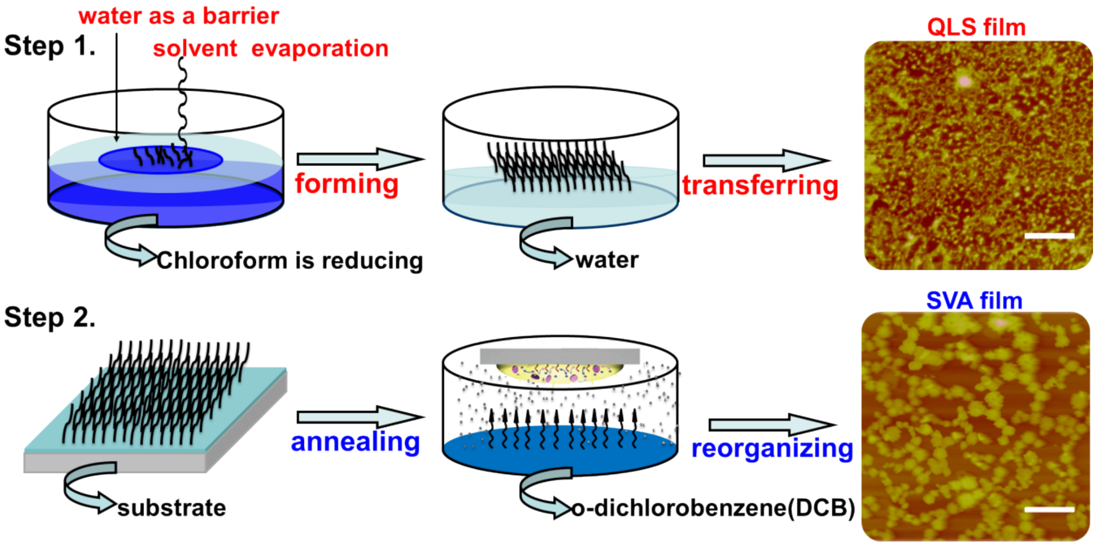

Fig. S8 The preparation procedure of QLS and SVA film. The scale bar in AFM images is 500 nm.

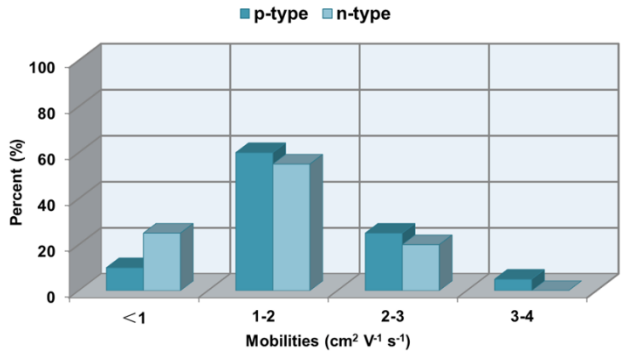

Fig. S9 The distribution diagram of devices mobilities.

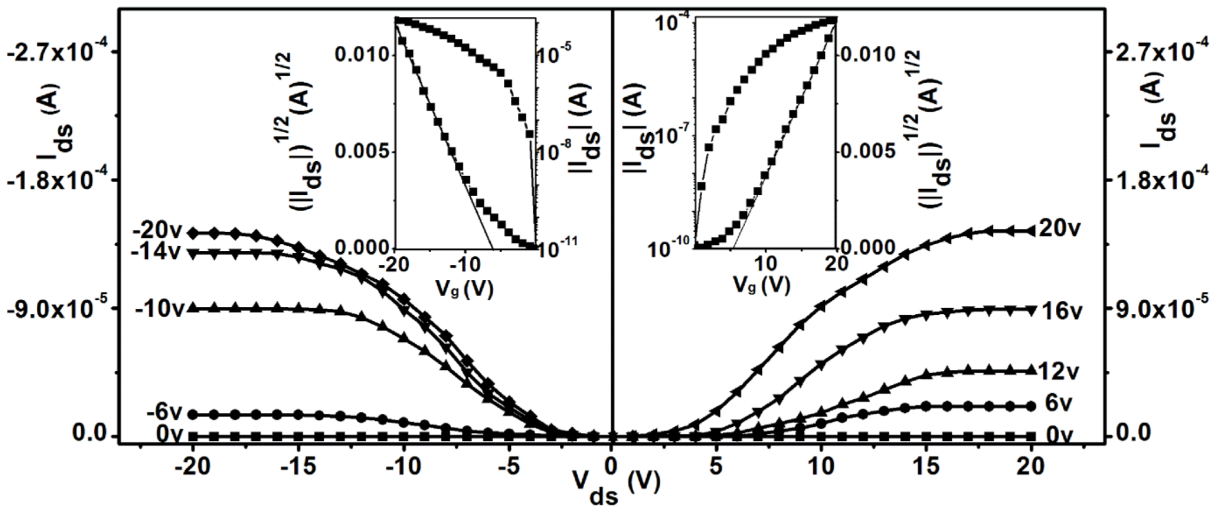

Fig. S10 Output characteristics (I_{ds} versus V_{ds}) and (insets) transfer characteristics ($|I_{ds}|^{1/2}$ versus V_G) of ambipolar OFET device based on the SVA films of (Pc)Eu[Pc(ONh)$_8$]Eu[Pc(ONh)$_8$] (**1**) deposited on HMDS-treated SiO_2/Si (300 nm) substrate with Au top contacts measured under ambient condition after two month storage in air.

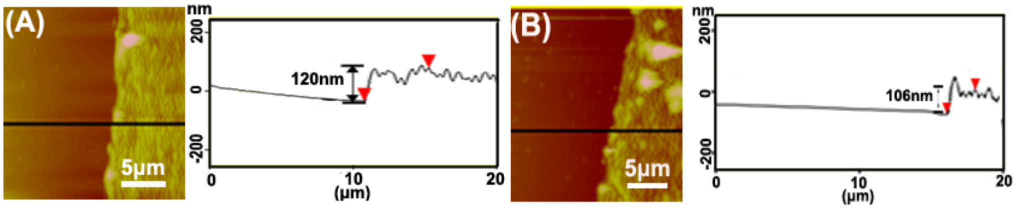

Fig. S11 AFM images for the thickness of the pristine QLS film (A) and SVA film (B) of **1** during measuring the depth of a scratch on the surface.

Table S1 Half-wave redox potentials (V *vs* SCE) in CH_2Cl_2 containing 0.1 mol dm^{-3} $[Bu_4N][ClO_4]$ together with the HOMO and LUMO levels of $(Pc)Eu[Pc(ONh)_8]Eu[Pc(ONh)_8]$ (**1**).

Compound	Oxd_3 /V	Oxd_2 /V	Oxd_1 /V	Red_1 /V	Red_2 /V	E_{HOMO} /eV[b]	E_{LUMO} /eV[b]	$\Delta E^o_{1/2}$ /V[a]
(**1**)	1.32	0.96	0.63	–0.44	–0.80	–5.07	–4.00	1.07

[a] $\Delta E^o_{1/2} = Oxd_1 - Red_1$, *i.e.* the HOMO − LUMO gap of corresponding molecule.

[b] Calculated from empirical formula of HOMO = – (Oxd_1 + 4.44 eV) and LUMO = – (Red_1 + 4.44 eV).

Table S2 The orientation angle of the phthalocyanine ring determined from polarized UV–vis absorbance of the films of (Pc)Eu[Pc(ONh)$_8$]Eu[Pc(ONh)$_8$] (**1**).

Sample	Film-type	$A_{//0°}$	$A_{\perp 0°}$	$D_{0°}$($A_{//}/A_{\perp}$)	$A_{//45°}$	$A_{\perp 45°}$	$D_{45°}$ ($A_{//}/A_{\perp}$)	θ°[a]
(**1**)	QLS film	0.191	0.191	1.00	0.203	0.198	1.03	52.6°
	SVA film	0.124	0.12	1.03	0.110	0.127	0.866	67.2°

[a] an orientation angle of Pc rings with respect to the substrate in the film can be calculated by the following equations:[17]

$$cos^2\theta = \frac{D_0 - (1 + D_0 sin^2\beta)D_\beta}{(1 - 2sin^2\beta)D_\beta - (1 + D_\beta sin^2\beta)D_0}$$

Where D is the dichroic ratio, $D_0 = A_{0//}/A_{0\perp}$, $D_\beta = A_{\beta//}/A_{\beta\perp}$, $A_{//}$ and $A_{\perp}$ are the absorbance of the film for polarized light with electric vectors parallel(//)and perpendicular(⊥) to the dipping direction, respectively. $\beta = sin^{-1}(sin\beta'/n)$, β' = 45°, n=1.148, is the refractive index of the film of **1**.

Table S3 The summary of the OFET device performances for both the SVA and the QLS films of (Pc)Eu[Pc(ONh)$_8$]Eu[Pc(ONh)$_8$] (**1**).

Sample	Film-type	μ_h ($cm^2 V^{-1} s^{-1}$)	I_{on}/I_{off}	V_{th}(V)	μ_e ($cm^2 V^{-1} s^{-1}$)	I_{on}/I_{off}	V_{th}(V)
(**1**)	QLS film	2.16×10^{-6}	~10^2	–16	3.15×10^{-6}	~10^2	2
	SVA film[a]	1.71	~10^6	–5	1.25	~10^6	9
	SVA film[b]	1.11	~10^6	–6	1.04	~10^6	6

[a] SVA film fabricated freshly; [b] SVA film stored in air after fabricated for two months.
